# Differences in the Elastomeric Behavior of Polyglycine-Rich Regions of Spidroin 1 and 2 Proteins

**DOI:** 10.3390/polym14235263

**Published:** 2022-12-02

**Authors:** Luis F. Pacios, Joseph Arguelles, Cheryl Y. Hayashi, Gustavo V. Guinea, Manuel Elices, Jose Perez-Rigueiro

**Affiliations:** 1 Departamento de Biotecnología-Biología Vegetal, ETSI Agronómica, Alimentaria y de Biosistemas, Universidad Politécnica de Madrid, 28040 Madrid, Spain; 2 Division of Invertebrate Zoology and Institute for Comparative Genomics, American Museum of Natural History, New York, NY 10024, USA; 3 Centro de Tecnología Biomédica (CTB), Universidad Politécnica de Madrid, 28223 Pozuelo de Alarcón (Madrid), Spain; 4 Departamento de Ciencia de Materiales, ETSI Caminos, Canales y Puertos, Universidad Politécnica de Madrid, 28040 Madrid, Spain; 5 Biomaterials and Regenerative Medicine Group, Instituto de Investigación Sanitaria del Hospital Clínico San Carlos (IdISSC), Calle Prof. Martín Lagos s/n, 28040 Madrid, Spain; 6 Centro de Investigación Biomédica en Red de Bioingeniería, Biomateriales y Nanomedicina (CIBER-BBN), Instituto de Salud Carlos III, 28029 Madrid, Spain

**Keywords:** silk, spidroin, molecular dynamics, elastomeric behavior

## Abstract

Two different polyglycine-rich fragments were selected as representatives of major ampullate gland spidroins (MaSp) 1 and 2 types, and their behavior in a water-saturated environment was simulated within the framework of molecular dynamics (MD). The selected fragments are found in the sequences of the proteins MaSp1a and MaSp2.2a of *Argiope aurantia* with respective lengths of 36 amino acids (MaSp1a) and 50 amino acids (MaSp2.2s). The simulation took the fully extended β-pleated conformation as reference, and MD was used to determine the equilibrium configuration in the absence of external forces. Subsequently, MD were employed to calculate the variation in the distance between the ends of the fragments when subjected to an increasing force. Both fragments show an elastomeric behavior that can be modeled as a freely jointed chain with links of comparable length, and a larger number of links in the spidroin 2 fragment. It is found, however, that the maximum recovery force recorded from the spidroin 2 peptide (F_max_ ≈ 400 pN) is found to be significantly larger than that of the spidroin 1 (F_max_ ≈ 250 pN). The increase in the recovery force of the spidroin 2 polyglycine-rich fragment may be correlated with the larger values observed in the strain at breaking of major ampullate silk fibers spun by Araneoidea species, which contain spidroin 2 proteins, compared to the material produced by spider species that lack these spidroins (RTA-clade).

## 1. Introduction

Spider silk constitutes a steady challenge for researchers studying biological materials within the framework of biomimetics [1,2,3,4]. In particular, the major ampullate gland silk (MAS) spun by spiders of the Araneomorphae lineage shows the largest work to fracture of any material [3], reaching a remarkable value of 500 MJ/m^3^ in the fibers spun by some Entelegynae species [5]. For comparison, such highly engineered polymeric materials as Kevlar yield a much modest value of 50 MJ/m^3^ [6].

The singular properties of MAS result from a process of almost 400 million years of evolution [7], in which spiders have diversified both the toolkit of silk glands [8] and the proteins, called spidroins [9], that make up the material [10]. The organization of spidroins follows a common design in which three regions may be distinguished that correspond to the N- and C-termini that flank a large, central core. The sequences of the N- and C-termini are highly conserved and play a critical role in the transition from protein solution to solid fiber [11,12], since they act as pH-switches that control the self-assembly of the spidroins in the gland. The central core is composed of a reduced set of small motifs that appear extensively repeated along this part of the sequence [13]. In this regard, the presence or absence of certain of these motifs in the central core is used to classify spidroins in a few types that include, among others, the major ampullate gland spidroin 1 (MaSp1) and the major ampullate gland spidroin 2 (MaSp2), which constitute the main components of major ampullate gland silk fibers. MaSp1 spidroins are defined by the presence of the sequence motifs –A_n_–, –GA–, and –GGX–. The presence of an additional –GPG– motif is characteristic of spidroins that belong to the MaSp2 family.

The appearance of the MaSp2 proteins seems to represent a major evolutionary breakthrough that is used to distinguish two different lineages within the Entelegynae [14]: RTA-clade and Araneoidea. Both lineages spin MAS fibers with comparable values of tensile strength (up to a true stress, σ, of σ ≈ 1500 MPa), but the silk produced by the Araneoidea tends to show larger values of strain at breaking (up to a true strain, ε, of ε ≈ 1.5), when compared with the maximum value of true strain, ε ≈ 0.5 attained by RTA-clade representatives [5]. The characterization of the influence of proline residues on the folding and native conformations of diverse proteins [15] was used to assign the increase in strain at breaking observed in araneoid MAS to the presence of MaSp2 proteins in their composition. This hypothesis was further supported by studies that found a clear correlation between this tensile parameter and the overall proline content in MAS fibers [16].

However, any attempt to establish a more profound relationship between spidroin 2 proteins and the tensile behavior of MAS beyond the qualitative correlation indicated above was hampered by two essential difficulties. To begin with, only partial sequences were available in the early works on the subject [9,13], so it was not possible to obtain a comprehensive knowledge of the polyglycine-rich regions that contain the –GGX–, –GA–, and –GPG– motifs. Additionally, the extreme variability of the tensile properties of MAS fibers [17,18] prevented a thorough comparison of the tensile properties of MAS fibers spun by different species.

The first problem was solved with the application of long-read sequencing techniques to retrieve the whole sequence of the genome of various spiders [19,20]. The second problem may be solved with the usage of the α* (alpha-star) alignment parameter [21,22] that allows to standardize the tensile properties of MAS silk spun by Entelegynae spiders following a very simple procedure. The combination of these technical advances led to the identification of certain features of the MaSp sequences that could account for the observed differences in the tensile properties of silk fibers spun by different species [23].

To gain a deeper insight into some fundamental aspects of the tensile behavior of spider silk fibers and its relationship with the composition and microstructure of the material, molecular dynamics (MD) appears as a remarkably powerful approach [24]. Together with experimental studies, MD-based computational approaches provide a valuable way to obtain dynamic features of biomolecular systems. Molecular dynamics has been applied to the study of biomolecules since the 1970s, and is frequently used in both chemistry and biology research both to interpret experimental results and to guide experimental work [25]. MD has been also an avenue for research in structure and properties of spider silk [26,27]. However, these and other MD studies have been focused on those aspects of the material related with the formation and dynamics of the nanocrystalline phase that appears in MAS as a result of the piling up of polyalanine motifs [6,28,29,30,31,32]. It might be argued that this tendency may originate from the limited amount of information that can be gained from the analysis of an arbitrary fragment of other regions different from the polyalanine runs, unless there is a thorough understanding of the whole sequence of the protein and of the detailed tensile properties of the MAS fibers.

This rationale is exploited in the present work by combining the sequencing of full-length spidroins from *Argiope aurantia* (black and yellow garden spiders) and the exhaustive mechanical characterization of the MAS fibers spun by this species within the framework of MD. Our atomic-level MD study addresses the distinct dynamic response to external forces of MAS fibers models with different amino acid composition. *A. aurantia* MAS was chosen since it exhibits the lowest value of the α* alignment parameter (α* = 0.0) of all characterized species at the writing of this work, and is used as reference to determine the values of the α* parameter of the other species. In addition, the whole set of major ampullate spidroins of this species is available and its analysis [23] indicates the existence of a high-level regularity in the organization of the polyglycine-rich regions located between consecutive polyalanine motifs. Consequently, one characteristic polyglycine-rich fragment of an MaSp1 representative and another characteristic polyglycine-rich fragment of an MaSp2 representative were selected and their tensile behavior in a water-saturated environment was simulated using MD calculations.

## 2. Materials and Methods

### 2.1. Sequence Selection

Two MaSp1 and seven MaSp2 genes are found in the genome of *A. aurantia*, as classified by the presence or not of the characteristic motif –GPG– in the sequence. Since no information is available at the time of writing this manuscript on the quantification of these proteins in MAS fibers and, consequently, on their contribution to the formation of MAS fibers, the spidroins MaSp1a and MaSp2.2a were selected as examples of each group due to the extreme regularity observed in the length of the polyglycine-rich regions between consecutive polyalanine motifs [23] in these two proteins.

**Figure 1:**
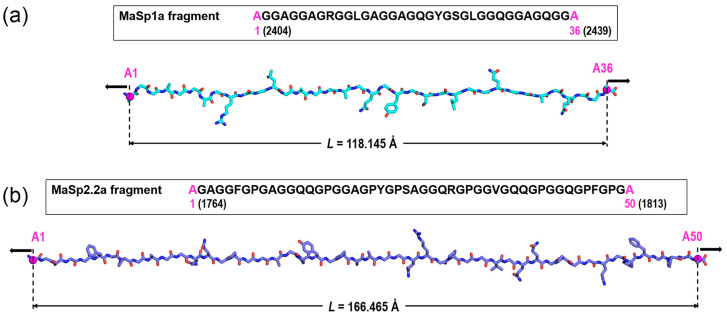
Sequence and extended β-pleated conformation of the selected fragments of *Argiope aurantia,* (**a**) MaSp1a (spidroin 1) and (**b**) MaSp2.2a (spidroin 2). Numbers in parentheses at both sequence ends indicate the corresponding residue numbers along the whole protein sequence. Distances are measured between both end Cα atoms (magenta spheres) in the optimized geometries. Black arrows represent the vector forces applied at both end Cα atoms.

Figure 1 shows the two selected fragments, one from the spidroin MaSp1a and another from the spidroin MaSp2.2a, on which the MD calculations were performed. The sequence of each fragment is indicated, as well as its geometry upon assumption of an extended β-pleated secondary structure as the initial reference conformation and after initial cycles of energy minimization to optimize the structure (see below). Both sequences are available from NCBI GenBank (Accession numbers MaSp1a (ON722379) and MaSp2.2a (ON722372), and the whole sequences are presented in Supplementary Figure S1. Each fragment corresponds to one of the largest regions among the set of repetitive fragments found in the central core of each selected spidroin. For consistency, both fragments start and end with an alanine residue that actually belongs to the adjacent polyalanine motifs found at the ends of each polyglycine-rich fragment. The MaSp1a fragment comprises 36 amino acids (including the two alanines at the ends) with a total length of 118.1 Å measured between both end Cα atoms in the optimized geometry of the completely extended β-pleated conformation, and the MaSp2.2a fragment comprises 50 amino acids with a total length of 166.5 Å in the same fully extended conformation.

### 2.2. Molecular Dynamics (MD) Calculations

Initial model geometries of MaSp1a and MaSp2.2a fragments were immersed in water, setting a solvation box with 24 Å margins in the elongated direction of the fragments and 18 Å margins in the perpendicular directions. Sodium and chloride ions were added to neutralize total charges while setting 0.150 M salt concentration. The whole molecular system for both fragments was generated from these solvation boxes under standard periodic boundary conditions. Geometries were initially optimized at 20,000 conjugate-gradient energy minimization steps with the NAMD 2.14 program [33]. After geometry optimization (which implicitly involves 0 K as only potential energy is minimized), MD equilibrations were run to raise temperature, heating water during 100 ps at 2 fs timesteps (50,000 equilibration steps) to reach a constant 298 K temperature. Production MD simulation runs were then performed for 20 ns at 2 fs timesteps (10 million steps) at constant P = 1 atm and T = 298 K upon different external forces applied as explained below. Simulation calculations numerically integrate Newton’s equation of motion for all atoms in the systems using the CHARMM 3.6 m [34] energy function, with mechanical (internal) forces leading to atom motions computed as the gradient of this energy. All these calculations were performed with the high-performance supercomputing version of the NAMD 2.14 program [33] in the Magerit supercomputer of the Universidad Politécnica de Madrid. The resulting all-atom numerical trajectories were saved at 1000 frames for processing and analysis with VMD 1.9.3 program [35].

To study the compared mechanical behavior of both MaSp1a and MaSp2.2a fragments through MD simulations, an external constant force was applied, pulling outward both end Cα atoms along the L direction (Figure 1). This external force is thus a constant drag vector defined on the L direction which is included in the equations of motion for every simulation run. The following applied external forces (in pN) were explored: 10, 20, 30, 40, 50, 100, 150, 200, 250, 300, 350, 400, 450, 500, 550, and 600. In addition, three independent MD simulations were run in the absence of external force to probe the intrinsic flexibility of both MaSp1a and MaSp2.2a fragments. This flexibility leads the extended chain to adopt irregular, compact conformations along the MD simulation, and different trajectories were then obtained to check for consistency. No extra simulations were performed under application of external forces, as the set of results obtained upon increasing the force for both L distance and RMSD values (Supplementary Table S1) provided consistent convergence. In all cases (with and without external forces), the simulations were run starting at the optimized structure of each fragment equilibrated at 298 K, as explained in the preceding paragraph.

## 3. Results

**Figure 2:**
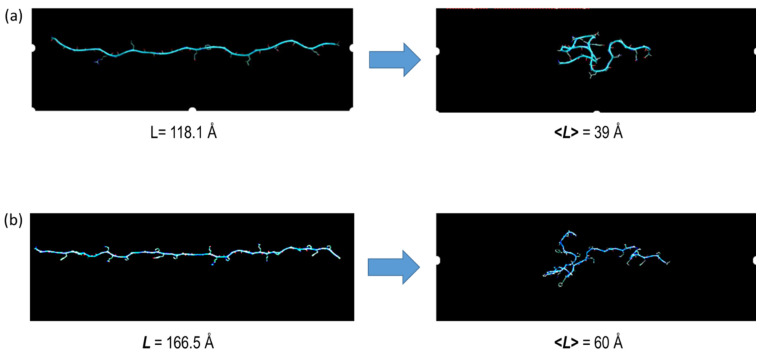
Comparison of the initial reference extended conformation (maximum L) and a representative conformation corresponding to the equilibrium state of the polyglycine-rich fragments of *A. aurantia,* (**a**) MaSp1a (spidroin 1) and (**b**) MaSp2.2a (spidroin 2), in MD simulations in the absence of external force. The distance between the end Cα atoms of the alanine residues, L (Figure 1), is indicated. The value <L> corresponds to the arithmetic mean of the distance L obtained from three different independent trajectories calculated for each fragment in the absence of external force (Table 1).

The initial structure of all the MD calculations on each fragment was the optimized reference extended β-pleated conformation equilibrated at 298 K. The distance between the Cα atoms at both ends, L (Figure 1) is thus the maximum length of each fragment. Then, the evolution of L was recorded over the 20 ns MD simulations in the absence and in the presence of external force. In all cases, equilibrium states were reached at different simulation times depending on the force applied. These states were assessed by following the evolution of the root mean square deviation (RMSD) computed for the superposition of backbone atoms in the structure of each trajectory frame with those in the initial structure. The stabilization of the RMSD variation in each trajectory was taken as a direct indication of equilibrium. In the absence of external forces, both MaSp1a and MaSp2.2a fragments tend to shrink dramatically, showing great variations of the RMSD with large standard deviations indicative of a considerable flexibility. This flexibility is manifested in large standard deviations of the L distance measured along the trajectory; a result found in three independent simulations of the fragments in the absence of external force (Table 1). The relationship between the initial reference conformation and the equilibrium conformation (reached at about 10 ns in the absence of external force in the three trajectories) is illustrated in Figure 2, where the arithmetic mean of the L distance (Table 1) is indicated. The evolution of the process is presented for one representative trajectory of each fragment as videos in Supplementary Materials Video S1.

Starting from the equilibrium structure indicated above, the simulations proceeded with the chain being subjected to an increasing force F exerted on the Cα of the alanines at the ends of each fragment. The average distance L computed for every trajectory showed longer average values and lower standard deviations as external forces increased (Supplementary Table S1). This is the expected result if one considers that an increasing force may provoke a decrease in the conformational freedom of the polypeptide chain. In addition, the RMSD values show, in all cases, lower average values and much lower standard deviations as external forces increased. Since the RMSD measures the difference between the structure at each trajectory frame and the initial structure (which corresponds to the fully elongated chains: see Figure 1 and Figure 2), lower RMSD values mean more similar structures. This result indicates that, as expected again, the fragments exhibit a rigidity that increases with greater external forces.

**Figure 3:**
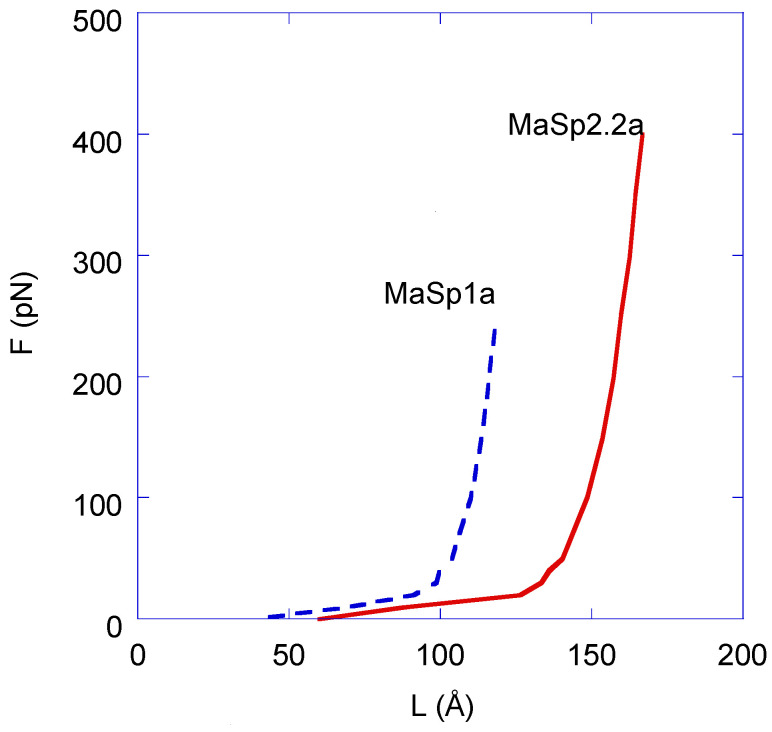
Force–distance curves obtained from the MD calculations on *A. aurantia* MaSp1a and MaSp2.2a fragments (values may be found in Supplementary Table S1).

These results are summarized in Figure 3, which plots the external force applied vs. the average distances L. The curves in Figure 3 start at the initial average equilibrium distance between the ends of the fragments in the absence of external force (39 Å for MaSp1a and 60 Å for MaSp2.2a; see Table 1) and extend to the maximum length of both fragments that correspond to the structures at which they are studied: the extended β-pleated conformation, since no other secondary structure geometry can have a longer length associated with a chain formed by peptide groups. The maximum 118.1 Å length for MaSp1a is reached at an applied external force of 250 pN, and the maximum 166.5 Å length for MaSp2.2a is reached at an applied external force of 400 pN (see Supplementary Table S1). It is assumed that the chain will break upon reaching its maximum length, as conventionally considered in the modeling of elastomers [36,37]. However, this breaking process necessarily involves the breaking of covalent bonds in the polypeptide chain and cannot therefore be modeled by MD calculations. In this regard, molecular dynamics is defined by the equations of motion of all the atoms in a molecular system (34,165 for MaSp1a-water and 45,432 for MaSp2.2a-water), so that space positions, velocities, forces, accelerations, and energies are computed for all the atoms at all the computational steps (10 million for each trajectory of each fragment in the current work) along the trajectory. The values of all these magnitudes are obtained by numerically integrating the equations of motions during the simulation time used for following the dynamics of the system, but MD is a classical, not quantum, mechanics framework, and formation or breaking of covalent bonds cannot then be addressed as both processes involve electron rearrangements only tractable through quantum methods.

From Figure 3 it may be concluded that the F–L curves of both fragments resemble the behavior of an elastomer since, in contrast to common materials (usually referred to as enthalpic materials in this context), the slope of the curves does not decrease. Since the simplest model of an elastomeric material is found in the freely jointed chain [38], it is natural to consider whether this model accounts for the curves presented above. The freely jointed chain models a macromolecule as a series of N links of given length, a (in the simplest case, all links are assumed to be equal). The links may rotate freely (i.e., no energy is involved in these rotations) around the sites connecting two consecutive links. The analysis of this system yields the following constitutive F–L equation, where L is the distance between the ends of the chain, k_B_ is Boltzmann constant, T is temperature, N is the number of links, and a is the length of a link:

(1) $$F=\frac{k_{B}T}{a}L{\left(Lucida\;calligraphy\right)}^{-1}\left(\frac{L}{Na}\right)$$

and $L\;{\left(Lucida\;calligraphy\right)}^{-1}$ is the inverse of Langevin’s function, defined as

(2) $$L\;\left(Lucida\;calligraphy\right)\left(x\right)=coth(x)-\frac{1}{x}$$

**Figure 4:**
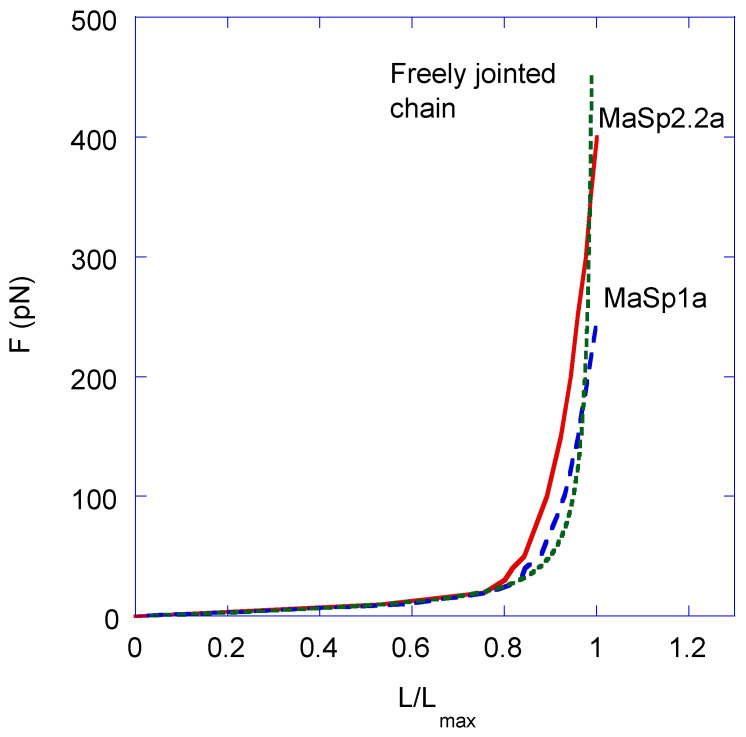
Force vs. L/L_max_ curves plotted with the data of the MD simulations (see Supplementary Table S1). The theoretical curve is calculated from Equation (1), assuming a length of the links of a = 8.1 Å and a temperature of T = 298 K.

In order to compare the experimental data with the values obtained applying the freely jointed chain, the F–L curves presented in Figure 3 are redrawn in Figure 4 by considering that (1) the independent variable is taken as L/Na (or, equivalently, L/L_max_ for each chain. L_max_ = 118.1 Å for the MaSp1a fragment and L_max_ = 166.5 Å for the MaSp2.2a fragment, since L_max_ = Na in each case), and (2) the chain reaches the minimum force of F = 0 pN at a value of the distance between Cα atoms of L = 0 Å. This latter condition takes into account that the freely jointed chain model considers all possible positions of the final link with respect to the initial link, so that the distance integrated along the three spatial directions averages to zero. In contrast, the MD calculations provide the standard deviation of this distance, which is always greater than zero. The practical difference between plotting the data taking as initial value L = 0 Å or L_equilibrium_ is almost negligible, since both values correspond to forces very close to F = 0 pN.

From Figure 4, it is apparent that the simulated curves are remarkably similar both qualitatively and quantitatively, albeit the MaSp2.2a fragment exhibits larger values of force at all values of L/L_max_. This difference is especially evident when comparing the maximum force reached by both fragments in the fully extended conformation: F_MaSp2.2a, max_ = 400 pN and F_MaSp1a, max_ = 250 pN. In this figure, the theoretical curve obtained by applying Equation (1) with a value of a = 8.1 Å is also represented. From this comparison, the excellent agreement between the theoretical curve and the curve obtained from the MD analysis of the MaSp1a fragment is apparent. The similarity between the theoretical curve and that obtained from the MD analysis of the MaSp2.2a fragment is also remarkable, although larger differences are found when comparing the numerical values of both curves.

Pursuing the application of the freely jointed chain model to the results obtained from the simulations, it is immediate to calculate the number of links of both fragments from the relationship

(3) $$L_{\max}=Na$$

by plugging into Equation (3) the value of a = 8.1 Å. Thus, the number of links of both fragments are N_MaSp1a_ ≈ 15 and N_MaSp2.2a_ ≈ 21.

## 4. Discussion

The identification of the elastomeric behavior of the MaSp1a and MaSp2.2a glycine-rich regions obtained from the molecular dynamics simulations when located in an environment with standard density of water molecules (at P = 1 atm and T = 298 K) may be considered as a first consistency test of the analysis. In effect, the study of the influence of water (or of a high-relative-humidity environment) on spider silk fibers has played a leading role in the understanding of this material since the discovery of supercontraction [39]. Supercontraction was initially defined by a significant reduction in the length of MAS fibers spun by orb-weaving spiders when immersed in water [40,41] and was found to be intimately connected with the appearance of elastomeric tensile properties when the material was tested under this condition [42,43]. Thus, the existence of a network of elastomeric chains was established as one of the three basic microstructural features that constitute the basic design elements of MAS fibers [36,37], along with β-nanocrystals [44,45] and an additional network of hydrogen bonds in the dry fiber that collapses when immersed in water [46].

In contrast to the immediate connection established between the formation of the β-nanocrystals and the polyalanine runs in the sequence of MaSp1 and MaSp2 spidroins [47], the assignment of the elastomeric behavior to the polyglycine-rich regions was much more intricate. In this regard, it was initially assumed that this phenomenon was restricted exclusively to the Araneoidea group within the Entelegynae [48] and, consequently, connected with the presence of the –GPG– motif in the spidroin 2 proteins expressed by the representatives of this group. This connection was supported by the finding of a correlation between the proline content of the MAS fibers and their mechanical behavior, in particular with their strain at breaking [16,49]. However, it was later found that some species outside of Araneoidea spin MAS fibers with the ability to supercontract [50,51], in spite of the absence of spidroin 2 proteins in these fibers. This finding was consistent with the identification of a number of conformational changes in the noncrystalline phase of MAS when the fibers were immersed in water, which pointed to the influence of alternative polyglycine-rich regions (i.e., regions that lack the –GPG– motif, such as YGGLGS(N)QGAGR) in the supercontraction of these fibers [52]. Subsequent studies [52,53,54,55] further supported the contribution of both MaSp1 and MaSp2 proteins to supercontraction. In particular, two distinct contributions to supercontraction in MAS fibers [56] were identified, and a clear correlation between the presence of tyrosine in the sequence and the emergence of supercontraction was established in biomimetic fibers [57].

In this context, molecular dynamics allows exploring the possible distinct roles of MaSp1 and MaSp2 proteins in supercontraction, as well as in the overall mechanical properties of MAS fibers. As shown above, the behavior of both fragments in terms of F vs. L/L_max_ plots is adequately described within the framework of the freely jointed chain model and, in particular, an excellent agreement between the molecular dynamics simulation and the theoretical analysis of the MaSp1a fragment is apparent from Figure 4. The concurrence with the theoretical elastomeric curve is poorer for the MaSp2.2a fragment quantitatively, although its qualitative behavior also corresponds to an elastomer. In both cases, the comparison of the theoretical analysis and the experimental results yields a common value for the length of the links of a = 8.1 Å and, correspondingly, a number of links of N_MaSp1a_ = 15 and N_MaSp2.2a_ = 21 for the MaSp1a and MaSp2.2a fragments.

The determination of the number of links and of the length of each link in both partial sequences allows checking the prediction obtained for the equilibrium distance, <L>, within the framework of the freely jointed chain model with the results retrieved from the simulation. In this regard, the freely jointed chain model establishes that the equilibrium distance between the ends of a chain formed by N links of length a is given by

(4) $$<L>=\sqrt{N}a$$

Plugging in the values indicated above, the model yields a value of <L_MaSp1a_> = 31.4 Å for the MaSp1a fragment, close to the mean value of <L> = 39 Å found in the molecular dynamics analysis. As noted above, the agreement between the equilibrium value calculated from the freely jointed chain model and the molecular dynamics simulation is less accurate for the MaSp2.2a fragment, for which a theoretical value of <L_MaSp2.2a_> = 37 Å is found.

The difference in the distance between the ends of each fragment at equilibrium, <L>, and the maximum length, L_max_, is especially relevant since it is related to the concept of “hidden length” [6,58] that is used to explain some aspects of the mechanical behavior of MAS fibers. The concept of “hidden length” is used to describe those parts of the spidroin chains that are not loaded at a given moment during tensile testing and, consequently, do not contribute to the tensile properties of the material at that instant. When the fiber is stretched, some regions, initially belonging to the “hidden length”, are removed from this phase and start to sustain load. The consideration of the elastomeric behavior of both poly-glycine-rich fragments allows interpreting the concept of “hidden length” from the equilibrium distance between the ends of the fragment at zero load, <L>, and the maximum length between those ends when the chain is fully extended, L_max_, as

(5) $$L_{hl}=L_{\max}-<L>$$

The application of this definition yields the values of L_hl_ = 79 Å and of L_hl_ = 106 Å for the MaSp1a and MaSp2.2a fragments, respectively. If these values are considered with respect to their contribution to the strain of the material, i.e., by dividing the absolute value of the hidden length, L_hl_, by the initial value of the equilibrium length for each fragment, <L>, both fragments yield comparable values of L_hl_/<L> = 2.0 (MaSp1a) and L_hl_/<L> = 1.8 (MaSp2.2a).

The similar values of “hidden length” found in both spidroins justify the importance of this concept in the understanding of the mechanical behavior of the material, but do not allow establishing any clear distinction between the influence of each polyglycine-rich fragment in this behavior. However, and as indicated in the Introduction, MAS spun by Araneoidea spiders, that are characterized by the presence of spidroin 2 proteins, exhibit larger values of strain at breaking than those spun by RTA-clade representatives that do not express proteins of this family [5].

In this context, molecular dynamics has been paramount to characterize biomolecular systems at atomic detail, to design and optimize experiments, to rationalize experimental observations, and to predict a large variety of properties. The continued progress, not only in the computational resources but also in the development and applications of new techniques and models, has led researchers to consider MD an invaluable methodological tool to study the biomolecular world. For a representative view of the current status of MD, the reader is referred, for instance, to the special issue in Ref. [59]. In the context of our work, the MD simulations clearly establish that the main difference between both fragments arises when comparing the maximum forces that each one may generate. In this regard, it is found that the MaSp2.2a fragment reaches a maximum force of F_max_ (MaSp2.2a) = 400 pN, significantly higher than the maximum value of F_max_ (MaSp1a) = 250 pN found in the MaSp1a fragment. The relevance of this difference in the maximum forces with respect to the higher strain at breaking of MAS fibers that contain the –GPG– motif may be established from the critical influence that the folding and unfolding of the spidroins exert on the tensile behavior of the material. In this regard, it can be assumed that these folding and unfolding processes proceed under the constraints imposed by those adjacent regions of the spidroin chains that form both the crystalline and noncrystalline phases of the material [60,61]. In particular, it was determined experimentally that an adequate conformational freedom of the chains is necessary for MAS fibers to reach their optimum mechanical performance [62].

Since the surrounding chains will hamper the conformational changes required by the proper mechanical performance of the fiber, either during stretching or during supercontraction, the existence of a potential “hidden length” does not imply that this parameter may be fully exploited to reach the optimum mechanical behavior of the material. In this context, the effective “hidden length” (i.e., the fraction of the potential “hidden length” that may contribute to the tensile properties of the material while tensile tested) will be increased by a corresponding increase in the recovery force exerted by the protein chains. Following this rationale, the observed increase in the recovery force of the MaSp2.2a fragment would extend the range of possible conformational modifications attainable to the protein within a dissipative environment. As indicated above, this increase in the effective “hidden length” allows justifying the larger strain at breaking of the MAS fibers spun by Araneoidea species in comparison with those spun by representatives of the RTA-clade.

## 5. Conclusions

The combination of a detailed genome sequencing and a thorough mechanical characterization of *Argiope aurantia* MAS silk allowed selecting two exemplars of the polyglycine-rich regions from each of the major ampullate spidroin 1 and spidroin 2 protein types. The deformation of both polyglycine-rich fragments in a water-saturated environment was analyzed through molecular dynamics calculations. The F–L curves calculated starting from the initial equilibrium configuration are shown to exhibit an elastomeric behavior that can be very accurately described by a freely jointed chain model with links of length a = 8.1 Å and a number of links N_MaSp1a_ = 15 for the MaSp1a fragment. A freely jointed model with parameters a = 8.1 Å and N_MaSp2.2a_ = 21 also accounts for the tensile behavior of the spidroin 2 fragment, although the quantitative agreement is poorer than that of the spidroin 1 fragment.

The molecular dynamics analysis establishes that the main difference between both fragments arises from the forces that the chain exerts at a given value of strain. In this regard, the MaSp2.2a fragment consistently shows larger values of force at any given strain and reaches a maximum value of F = 400 pN when the chain is fully extended. This value may be compared to the maximum value of F = 250 N found for the fully extended spidroin 1 fragment. It is hypothesized that a larger recovery force in spidroin 2 polyglycine-rich regions would allow an increase in the length of the chain that can sustain conformational changes in the presence of the other protein chains that form the fiber. This increase in the “effective hidden length”, in turn, allows justifying the increase in the strain at breaking observed in the MAS fibers of araneoid spiders that contain spidroin 2 proteins in their composition, in contrast with the MAS fibers of RTA-clade representatives that do not express this type of spidroins.

## Figures and Tables

**Table 1:** Average values of the distance between the Cα of the residues at the ends of the fragments, L (Figure 1), and their corresponding standard deviations for three independent MD trajectories of MaSp1a and MaSp2.2a in the absence of external force. The arithmetic mean and standard deviation calculated from these trajectory values for each fragment is indicated in the last row.

	MaSp1a		MaSp2.2a	
	Average (Å)	Std. Deviation (Å)	Average (Å)	Std. Deviation (Å)
Trajectory 1	40.1	14.6	52.3	27.6
Trajectory 2	33.4	20.5	60.1	22.0
Trajectory 3	43.9	15.1	68.8	24.8
Mean	39 ± 3 Å		60 ± 5 Å	

## Data Availability

Data are available upon request to the corresponding author.

## Supplementary Materials

The following supporting information can be downloaded at: https://www.mdpi.com/article/10.3390/polym14235263/s1, Table S1: Summary of the average values and standard deviations of the geometrical parameters obtained in the MD simulation; Figure S1: Sequences of the proteins used for the MD simulation; Supplementary Materials Video S1 (two videos): Examples of the trajectories of MaSp1a and MaSp2.2a protein fragments in the dynamical evolution from initial reference to equilibrium conformations.
