# Supplementary material for: Differences in the Elastomeric Behavior of Polyglycine-Rich Regions of Spidroin 1 and 2 Proteins
Source: Polymers (Basel). 2022 Dec 2;14(23):5263. doi: 10.3390/polym14235263 (PMC9738160; doi:10.3390/polym14235263)
Supplement: Supplementary file 1 [file polymers-14-05263-s001.zip › Supplementary Figure S1.pdf]

>Aaur_MaSp1a
MNWTTRLALSVLVVICSQSIFALGQSPWQSASMAESFMTYFSAALGQSGAFTNEQMDDIDTIATSIKMGV
DKMERSGKTSQNKLQAMNMAFASAVAEIAIAEGGGQSAQVKTNAIADALASAFLQTTGVVNRQFINEIRG
LISMFAQANSISSSSGYASASAEAAAGSAGGAGQGYGAGLGGQGGAGQGGAAAAAAAAGGQGGQGGYGGL
GSQGAGQGGYGAGQGGAGAAAAAAAAGGAGGAGRGGLGAGGGGQGYGSGLGGQGGAGQGGAAAAAAAAGG
QGGQGGYGGLGSQGAGQGGYGAGQGGAGAAAAAAAAGGAGGAGRGGLGAGGAGQGYGSGLGGQGGAGQGG
AAAAAAAAGGQGGQGGYGGLGSQGAGQGGAGAAAAAAAAGGAGGAGRGGLGAGGAGQGYGSGLGGQGGAG
GGAAAAAAAAGGQGGQGGYGGLGSQGASQGGAGRGAAAAAAAAGGQGGQGGYGGLGSQGAGQGGYGAGQG
GAGAAAAAAAAGGTGGAGRGGLGAGGAGQGYGSGLGGQGGAGQGGAAAAAAAAGGQGGLGGYGGLGSQGA
GQGGYGAGQGGAGAAAAAAAAGGAGGAGRGGLGAGGAGQGYGSGLGGQGGAGQGGAAAAAAAAGSQGGQG
GYGGLGSQGAGQGGYGAGQGGAGAAAAAAAAGGAGRGGLGAGGAGQGYGSGLGGQGGAGGGAAAAAAAAG
GQGGQGGYGGLGSQSAGQVGAGRGAAAAAAAAGGQGGQGGYGGLGSQGAGQGGYGAGQGGAGAAAAAAAA
GGAGGAGRGGLGAGGAGQGYGSGLGGQGGAGQGGAAAAAAAAGGQGGQGGYGGLGSQGAGQGGYGAGQRG
AGAAAAAAAAGGAGGAGRGGLGAGGAGQGYGSGLGGQGGAGQGGAAAAAAAAGDQGGQGGYGGLGSQGAG
PGGYGAGQGGAGAAAAAAAAGGAGRGGLGAGGAGQGYGSGLGGQGGAGQGGAAAAAAASGGQGGQGGYGG
LGSQGAGQGGYGAGAAAAAAAAGGAGRGGLGAGGAGQGYGSGLGGQGGARGGAAAAAAAASGQGGQGGYG
GLGSQGAGQGGAGRGASAAAAAAGGQGGQGGYGGLGSQGAGQGGYGAGQGGAGAAAAAAADGGSGGAGRG
GLGAGGAGRYGSGLGGQGGDGQGGAAAAAAAAAGGQSGQGGYGGLGSQGAGQGGYGAGQGGAGAAAAAAA
AGGAGGAGRGGLGAGGAGQGYGSRLGGQGRAGQGGAAAAAAAAGGQGGQGGYGGLGSQGAGQGGYGAGQG
GAGAAAAAAAAGAAGGAGRGGLGAGGAGQGYGSGLGGQGGAGQGGAAAAAAAAGGQGGQGGYGGLGSQGA
GQGGYGAGQGGAGAAAAAAAAGGAGGAGRGGLGAGGAGRYGSGLGGQGGDGQGGAAAAAAAAAGGQGGYG
GLGSQGAGQGGAGRGAAAAAAAAGGQGGQGGYGGLGSQGAGQGGYGAGQGGAGAAAAAAAAGGAGGAGRG
GLGAGGAGQGYGSGLGGQGGAGGGAAAAAAAAGGQGGQGGYGGLGSQGAGQGGAGRGASAAAAAAGGQGG
QGGYGRLGSQGAGQGGYGAGQGGAGAAAAAGGAGGAGRGGLGAGGAGRYGSGLGGQGGDGQGGAAAAAA
AAAGGQGGYGGLGSQGVGQGGAGRGAAAAAAAAGGQGGQGGYGGLGSQGAGQGGYGAGQGGAGAAAAAAA
AGGAGGAGRGGLGAGGAGRYGSGLGGQGGAGQGGAAAAAAAAGGQGGQGGYGGLGSQGAGQGGYGAGAAA
AAAAAGGAGGAGRGGLGAGGAGQGYGSGLGGQGGAGQGGAAAAAAAAGGQGGQGGYGGLGSQGAGQGGYG
AGQGGAGAAAAAAAAGGAGGAGRGGLGAGGAGRYGSGLGGQGGDGQGGAAAAAAAAAGGQGGQGGYGGLG
SQGAGQGGAGRGAAAAAAAAGGQGGQGGYGGLGSQGAGQGGYGAGQGGAGAAAAAAAAGGAGGAGRGGIG
AGGAGRYGSGLGGQGGAGQGGAAAAAAAAGGQGGQGGYGGLGSQGAGQGGYGAGAAAAAAAAGGAGGAGR
GGLGAGGAGQGYGSGLGGQGGAGQGGAAAAAAAAGGQGGQGGYGGLGSQGAGQGGYGAGQGGAGAAAAAA
AAGGAGGAGRGGLGAGGAGQGYGSGLGGQGGAGQGGAAAAAAAAAGGQGGQGGYGGLGSQGAGQGGYGAG
QGGAGAAAAAGGAGGAGRGGLGAGGAGQGYGSGLGGQGGAGQGGAAAAAAAAGGQGGQGGYGGLGSEGAG
QGGYGAGQGGAGAAAAAAAAGGAGGAGRGGLGAGGAGQGYGSGLGGQGGAGQGGAAAAAAAAGGQGGQGG
YGGLGSQGAGQGGAGRGAAAAAAAAGGQGGQGGYGGLGSQGAGQGGYGAGQGGAGAAAAAAAAGGAGGAG
RGGLGAGGAGQGYGSGLGGQGGAGQGGAAAAAAAAGGQGGQGGYGGLGSQGAGQGGYGAGQGGAGAAAAA
ATAGGAGGAGRGGLGAGGAGQGYGSGLGGQGGAGQGGAAAAAAAAGGQGGQGGYGGLGSQGAGQGGYGAG
AAAAAAAAGGAGGAGRGGLGAGGAGQGYGSGLGGQGGAGQGGAAAAAAAAGGQGGQGGYGGLGSQGAGQG
GYGAGQGGAGAAAAAAAAGGAGGAGRGGLGAGGAGRYGSGLGGQGGDGQGGAAAAAAAAAGGQGGQGGYG
GLGSQGVGRDAAAAAAAAGGQGGQGGYGGLGSQGAGQGGYGAGQGGAGAAAAAAAAGGAGGAGRGGLGAG
GAGQGYGSGLGGQGGAGQGGAAAAAAAAAGGQGGQGGYGGLGSQGAGQGGYGVGQRGAGAAAAAAAAGGA
GGAGRGGLGAGGAGQVYGSGLGGQGGAGQGGAATAAAAAGGQGGQGGYGGLGSEGAGQGGYGAGQGGAGA
AAAAAAAGGAGGAGRGGLGAGGAGQGYGSGLGGQGGAGQGGAAAAAAAAGGQGGQGGYGGLGSQGAGQGG
AGRGAAAAAAAAGGQGGQGGYGGLGSQGAVQGGAGRGAAAAAAAAGGQGGQGGYGGLGSQGAGQGGAGRG
AAAAAAAAGGQGGQGGYGGLGSEGAGQGGAGRGAAAAAAAAGGQGGQGGYGGLGSQGAGQGGYGAGQGGA
RAAAAAAAAGGAGGAGRGGLGAGGAGQGYGSGLGGQGGAGQGGAAAAAAAAAAGGQGGQGGYGGLGSQGA
GQGGYGAGQGGAGAAAAAATAGGAGGAGRGGLGAGGAGQGYGSGLGGQGGAGQGGAAAAAAAAGGQGGQG
GFGRFSSQEAGQGAYGGGAYSGQQGAAASVSAASAAASRLSSPGAASRVSSAVTSLVSSGGPTNPAALSN
TISNVVSQISESNPGLSGCDVLVQALLELVSALVHILGSANIGQVNSSAAGQSASLVRQSVYQALS

>Aaur_MaSp2.2a
MNWSIRLALLGFVVLSTQTIFAAGQAATPWENTQLAEDFIISFLRFIGQSGAFSPDQLDDMSTIGETLKTAIEKMAQ
SRKSSKSKLQALNMAFASSMAEIAVAEKGGLSLEAKTNAIANALASAFLETTGFVNQQFVSEIKSLIYMIAQASANE
ISGSAAAAGGGSGGFGSGQGGYGQGAYASASAASAYGSAPQGAGGPAPQGLSQQGPVRQGPYGPSAAVAATAVGGRP
QGRSASSQQGPSQQGPYGPGAAGAAAAAGGYGPGVGQQGPGDAGQQGPYGPGAAAVGGYGPGARAGGPQRPIGAGPS
LPSARGPQGPGGSGPGSQGPFEPAAAAAAAAAARGFGPGASGQKGPGEAGQQGPGGAGQQGPGGQGLFGPGAAAAAA
AAAGGFGPGAGGQRGPGQQGPGGQGPSGPGAAAAAAAAAGGFGPGGAGAGPQAGQRGPGGAGAGAAAAAAAGAGGFG
PGAGGQQGPGGAGPYGPSAGGQRGPGGVGQQGPGGQGPFGPGAAAAAAAAAGGFGPGGAGVGPQAAPGQQGPGGAGP
YGPGAAAAAAAAGGFGPGAGGQRGPGQQGLFGPGAAAAAAAAAGGFGPGAGGQKVPGGAGQQGPGGQGPYGPGAAAA
AAAAGGFGPGAGGQRGPGQQGPGGQGPSGPGAAAAAAAAAAGGFGPGGAGAGPQAGQRGPGGAGAGAAAAAAAGAGG
FGPGAGGQQGPGGAGPYGPSAGGQRGPGGVGQQGPGGQGPFGPGAAAAAAAAAGGFGPGGAGVGPQAAPGQQGPGGA
GPYGPGAAAAAAAAAAGGFGPGAGGQRGPGQQGLFGPGAAAAAAAAAGGFGPGAGGQKVPGGAGQQGPGGQGPYGPG
AAAAAAAAGGFGPGAGGQRGPGQQGPGGQGPSGPGAAAAAAAAAAGGFGPGGAGAGPQAGQRGPGGAGAGAAAAAAA
GAGGFGPGAGGQQGPGGAGPYGPSAGGQRGPGGVGQQGPGGQGPFGPGAAAAAAAAAGGFGPGGAGVGPQAAPGQQG
PGGAGPYGPGAAAAAAAAAAGGFGPGAGGQRGPGQQGLFGPGAAAAAAAAAGGFGPGAGGQKVPGGAGQQGPGGQGP
YGPGAAAAAAAAGGFGPGAGGQRGPGQQGPGGQGPSGPGAAAAAAAAAAGGFGPGGAGAGPQAGQRGPGGAGAGAAA
AAAAGAGGFGPGAGGQQGPGGAGPYGPSAGGQRGPGGVGQQGPGGQGPFGPGAAAAAAAAAGGFGPGGAGVGPQAAP
GQQGPGGAGPYGPGAAAAAAAAAAGGFGPGAGGQRGPGQQGLFGPGAAAAAAAAAGGFGPGAGGXKVPGGAGQQGPG
GQGPYGPGAAAAAAAAGGFGPGAGGQRGPGQQGPGGQGPSGPGAAAAAAAAAAGGFGPGGAGAGPQAGQRGPGGAGA
GAAAAAAAGAGGFGPGAGGQQGPGGAGPYGPSAGGQRGPGGVGQQGPGGQGPFGPGAAAAAAAAAGGFGPGGAGVGP
QAAPGQQGPGGAGPYGPGAAAAAAAAGGFGPGAGGQRGPGQQGLFGPGAAAAAAAAAGGFGPGAGGXKVPGGAGQQG
PGGQGPYGPGAAAAAAAAGGFGPGAGGQRGPGQQGPGGQGPSGPGAAAAAAAAAAGGFGPGGAGAGPQAGQRGPGGA
GAGAAAAAAAGAGGFGPGAGGQQGPGGAGPYGPSAGGQRGPGGVGQQGPGGQGPFGPGAAAAAAAAAGGFGPGGAGV
GPQAAPGQQGPGGAGPYGPGAAAAAAAAAAGGFGPGAGGQRGPGQQGLFGPGAAAAAAAAAGGFGPGAGGXKVPGGA
GQQGPGGQGPYGPGAAAAAAAAGGFGPGAGGQRGPGQQGPGGQGPSGPGAAAAAAAAAAGGFGPGGAGAGPQAGQRG
PGGAGAGAAAAAAAGAGGFGPGAGGQQGPGGAGPYGPSAGGQRGPGGVGQQGPGGQGPFGPGAAAAAAAAAGGFGPG
GAGVGPQAAPGQQGPGGAGPYGPGAAAAAAAAAAGGFGPGAGGQRGPGQQGLFGPGAAAAAAAAAGGFGPGAGGQKV
PGGAGQQGPGGQGPYGPGAAAAAAAAGGFGPGAGGQRGPGQQGPGGQGPSGPGAAAAAAAAAAGGFGPGGAGAGPQA
GQRGPGGAGAGAAAAAAAGAGGFGPGAGGQQGPGGAGPYGPSAGGQRGPGGVGQQGPGGQGPFGPGAAAAAAAAAGG
FGPGGAGVGPQAAPGQQGPGGAGPYGPGAAAAAAAAAAGGFGPGAGGQRGPGQQGLFGPGAAAAAAAAAGGFGPGAG
GQKVPGGAGQQGPGGQGPYGPGAAAAAAAAGGFGPGAGGQRGPGQQGPGGQGPSGPGAAAAAAAAAAGGFGPGGAGA
GPQAGQRGPGGAGAGAAAAAAAGAGGFGPGAGGQQGPGGAGPYGPSAGGQRGPGGVGQQGPGGQGPFGPGAAAAAAA
AAGGFGPGGAGVGPQAAPGQQGPGGAGPYGPGAAAAAAAAAAGGFGPGAGGQRGPGQQGLFGPGAAAAAAAAAGGFG
PGAGGQKVPGGAGQQGPGGQGPYGPGAAAAAAAAGGFGPGAGGQRGPGQQGPGGQGPSGPGAAAAAAAAAAGGFGPG
GAGAGPQAGQRGPGGAGAGAAAAAAAGAGGFGPGAGGQQGPGGAGPYGPSAGGQRGPGGVGQQGPGGQGPFGPGAAA
AAAAAAGGFGPGGAGVGPQAAPGQQGPGGAGPYGPGAAAAAAAAAAGGFGPGAGGQRGPGQQGLFGPGAAAAAAAAA
GGFGPGAGGQKVPGGAGQQGPGGQGPYGPGAAAAAAAAGGFGPGAGGQRGPGQQGPGGQGPSGPGAAAAAAAAAAGG
FGPGGAGAGPQAGQRGPGGAGAGAAAAAAAGAGGFGPGAGGQQGPGGAGPYGPSAGGQRGPGGVGQQGPGGQGPFGP
GAAAAAAAAAGGFGPGGAGVGPQAAPGQQGPGGAGPYGPGAAAAAAAAAAGGFGPGAGGQRGPGQQGLFGPGAAAAA
AAAAGGFGPGAGGQKVPGGAGQQGPGGQGPYGPGAAAAAAAAGGFGPGAGGQRGPGQQGPGGQGPSGPGAAAAAAAA
AAGGFGPGGAGAGPQAGQRGPGGAGAGAAAAAAAGAGGFGPGAGGQQGPGGAGPYGPSAGGQRGPGGVGQQGPGGQG
PFGPGAAAAAAAAAGGFGPGGAGVGPQAAPGQQGPGGAGPYGPGAAAAAAAAAAGGFGPGAGGQRGPGQQGLFGPGA
AAAAAAAAGGFGPGAGGQKVPGGAGQQGPGGQGPYGPGAAAAAAAAGGFGPGAGGQRGPGQQGPGGQGPSGPGAAAA
AAAAAAGGFGPGGAGAGPQAGQRGPGGAGAGAAAAAAAGAGGFGPGAGGQQGPGGAGPYGPSAGGQRGPGGVGQQGP
GGQGPFGPGAAAAAAAAAGGFGPGGAGVGPQAAPGQQGPGGAGPYGPGAAAAAAAAAAGGFGPGAGGQRGPGQQGLF
GPGAAAAAAAAAGGFGPGAGGQKVPGGAGQQGPGGQGPYGPGAAAAAAAAGGFGPGAGGQRGPGQQGPGGQGPSGPG
AAAAAAAAAAGGFGPGGAGAGPQAGQRGPGGAGAGAAAAAAAGAGGFGPGAGGQQGPGGAGPYGPSAGGQRGPGGVG
QQGPGGQGPFGPGAAAAAAAAAGGFGPGGAGVGPQAAPGQQGPGGAGPYGPGAAAAAAAAGGFGPGAGGQRGPGQQG
PGGQGLFGPGAAAAAAAAAGGFGPGAGGQKGPGGAGQQGPGGQGPYGPGAAAAAAAAGGFGPGTGGQRGPGQQVPGG
QGPSGPGAAAAAAAAAGGFGPGGAGPGPKAGQGGARFYRPGAAVATAAVGGYGPGAGQQGPAAPSQQGPGRQIPYGP
GAAAAVVGVYAPVPQRPTASAAASRLASPEASSRVSSAVSSLVSSGPTNPAALSNTISSVVSQISASNPGLSGCDVL
VQALLEIVSALVHILGYSSIGQINYGAASQYARLVGQSVAQALG
