# Supplementary material for: Differences in the Elastomeric Behavior of Polyglycine-Rich Regions of Spidroin 1 and 2 Proteins
Source: Polymers (Basel). 2022 Dec 2;14(23):5263. doi: 10.3390/polym14235263 (PMC9738160; doi:10.3390/polym14235263)
Supplement: Supplementary file 1 [file polymers-14-05263-s001.zip › Supplementary Material Video S1.pptx]

## Slide 1
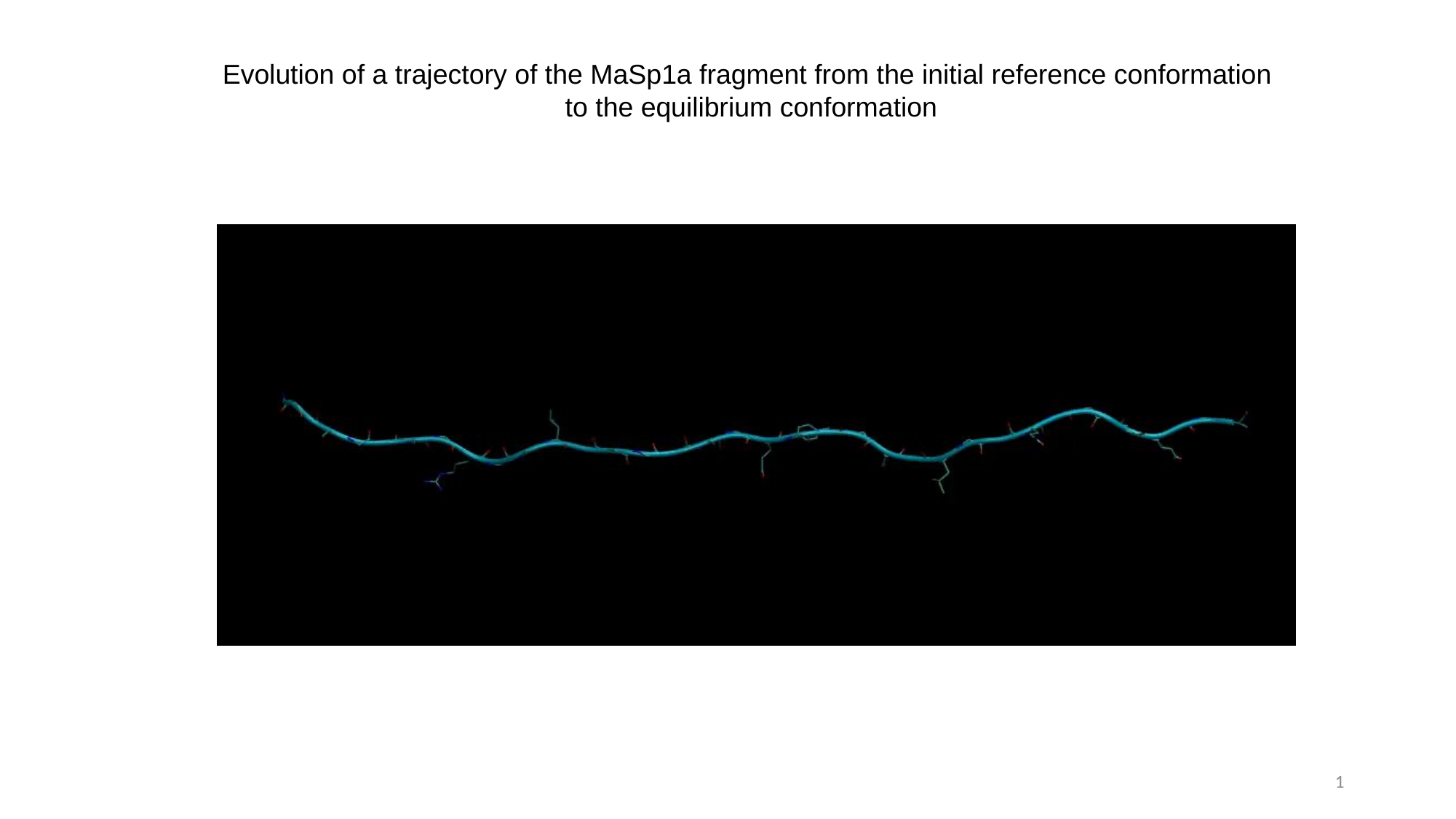

Evolution of a trajectory of the MaSp1a fragment from the initial reference conformation
to the equilibrium conformation
1

## Slide 2
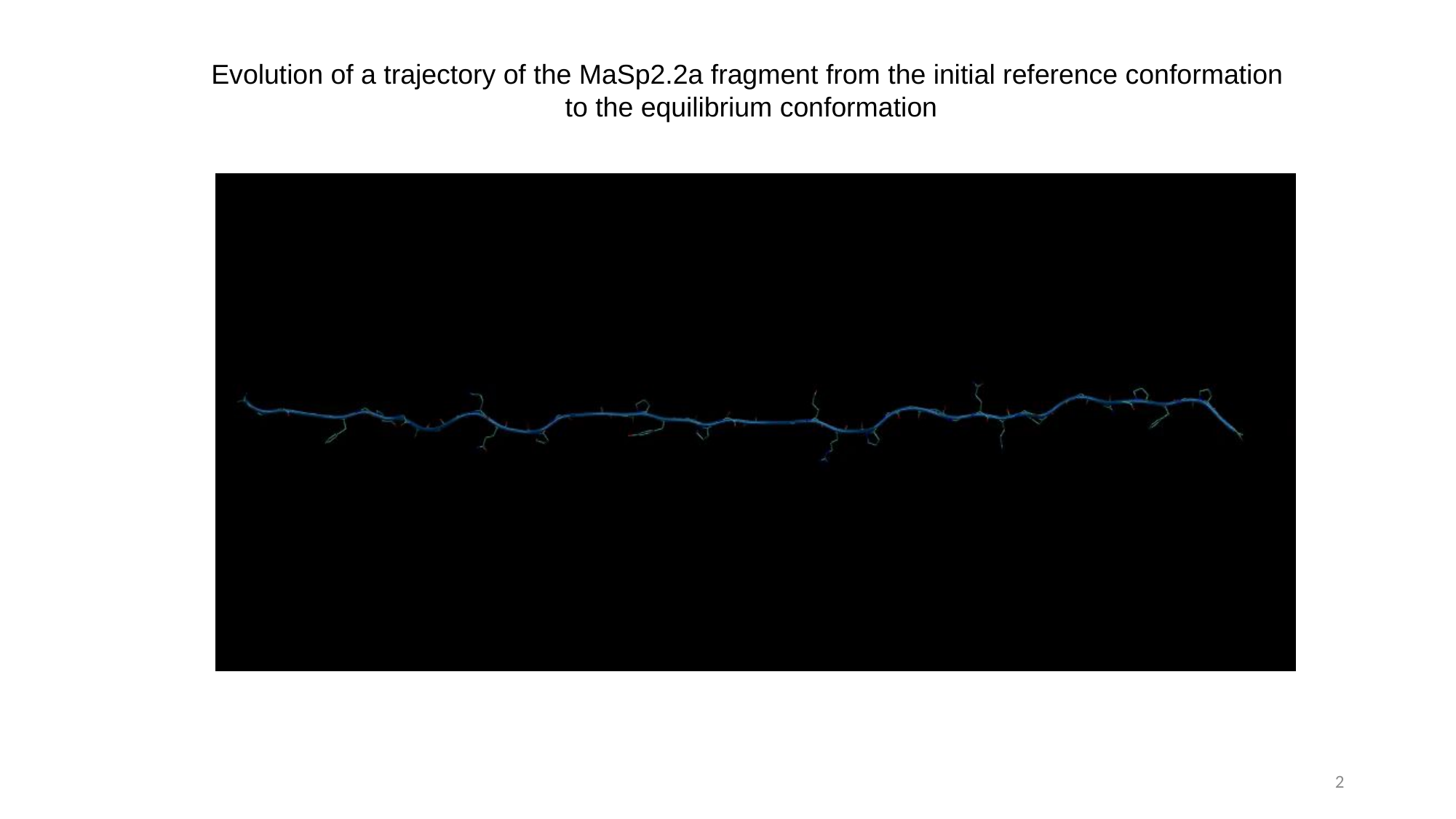

Evolution of a trajectory of the MaSp2.2a fragment from the initial reference conformation
to the equilibrium conformation
2
