# Supplementary material for: Differences in the Elastomeric Behavior of Polyglycine-Rich Regions of Spidroin 1 and 2 Proteins
Source: Polymers (Basel). 2022 Dec 2;14(23):5263. doi: 10.3390/polym14235263 (PMC9738160; doi:10.3390/polym14235263)
Supplement: Supplementary file 1 [file polymers-14-05263-s001.zip › Supp_Table_S1.pdf]

Supplementary Table S1. Average values and standard deviations of: (*a*) Root Mean Square Deviation (RMSD) computed for backbone atoms in the superposition of the structure of each trajectory frame and the initial structure, and (*b*) distance L measured between Cα atoms of end alanines in MaSp1a and MaSp2.2a fragments in MD simulations upon external forces ***F*** applied. In the absence of forces, results for three independent trajectories (*traj.n*, *n*=1,2,3) are included. Last entries in the Table correspond to the maximum elongation L of the fully extended conformation of both MaSp1a and MaSp2.2a polypeptide chains.

	**MaSp1a**					**MaSp2.2a**			
F (pN)	RMSD (Å)		L (Å)			RMSD (Å)		L (Å)	
	Average	Std.dev.	Average	Std.dev.		Average	Std.dev.	Average	Std.dev.
0 (*traj.1*)	24.0	5.47	40.08	14.6		29.6	7.13	52.28	27.6
0 (*traj.2*)	26.0	5.94	33.37	20.5		28.5	6.32	60.06	22.0
0 (*traj.3*)	20.6	4.44	43.88	15.1		26.5	6.99	68.77	24.8
10	14.5	3.14	69.35	11.2		22.3	4.34	88.52	14.5
20	8.43	1.97	90.86	6.33		11.5	2.39	126.0	6.64
30	5.97	1.55	98.58	4.91		9.24	1.79	133.3	4.76
40	5.72	1.30	99.72	4.40		7.93	1.37	135.9	5.13
50	4.50	0.97	103.6	2.93		7.67	1.27	140.3	3.27
100	3.50	0.56	109.9	1.90		5.33	0.74	148.4	2.17
150	2.71	0.45	113.3	1.66		4.20	0.59	153.5	1.96
200	2.56	0.37	115.9	1.33		3.32	0.53	157.1	1.76
250	2.12	0.24	118.1	1.22		3.09	0.47	159.4	1.72
300						2.73	0.38	162.4	1.42
350						2.42	0.39	164.3	1.79
400						2.00	0.28	166.5	1.36
